# Comparison of health related quality of life of primary school deaf children with and without motor impairment

**DOI:** 10.1186/1824-7288-36-75

**Published:** 2010-11-12

**Authors:** Venkadesan Rajendran, Finita Glory Roy

**Affiliations:** 1 Institute of Rehabilitation Science, Holy Cross College affiliated to Bharathidasan University, Trichy, Tamilnadu, India

## Abstract

**Objective:**

To compare the health-related quality of life (HRQOL) of primary school-age deaf children with or without motor impairment to that of typically developing peers.

**Methods:**

This study was a prospective, cross sectional study. With age-matched controls, 100 children were analyzed in each of the following three categories: normal hearing, hearing impaired without motor impairment, and hearing impairment with motor impairment. The Paediatric Quality of Life Inventory (PedsQL™) version 4.0 was used to assess the HRQOL.

**Results:**

Hearing impairment along with motor impairment in children is associated with significantly increased proportions of suboptimal levels of function and significantly lower HRQOL. Children with hearing impairment and no motor impairment had significantly lower scores in the emotional health and school function domains of the PedsQL than children with normal hearing, but there was no significant difference in the physical and social health domain scores. Children with hearing impairment and motor impairment showed significantly lower scores in all domains of the PedsQL compared to children with normal hearing. Scores in all four domains of the PedsQL differed between children with hearing impairment and no motor impairment and children with hearing impairment and motor impairment.

**Conclusion:**

These findings indicate that children with hearing impairment, both with and without motor impairment, have a diminished health-related quality of life.

## 1. Introduction

Health related quality of life (HRQOL) is a multidimensional construct, consisting at the minimum of physical and psychosocial health dimensions (including emotional and cognitive) delineated by the WHO; it could be assessed by both objective and subjective means [1-5]. Hearing loss is the third most common congenital and acquired disease in children that leads to major serious health implications, which affects 1 to 3 children/1000 [6]. Childhood deafness often causes psycho-intellectual and social developmental disorders in children because they have difficulty interacting with their surroundings [7].

For decades emphasis has been placed on the implementation of screening services for children suffering from hearing loss. While hearing tests provide information regarding the status of the individual's hearing loss, they fail to provide data on the impact of hearing loss on the child's social and emotional life, physical abilities, and academic performance. Children with hearing loss may be subject to developmental delays. Most studies emphasize speech impairment and fail to address functional, neuro-developmental and behavioral outcomes. In the twenty-first century, health-related quality of life (HRQOL) is an essential outcome measure in clinical trials and healthcare. One of the important changes in healthcare in recent years has been the shift toward assessment of health status and outcome, and the importance of HRQOL in measuring the health of individuals and populations is increasingly recognized [8].

Quantitative measures of the effects of hearing loss on HRQOL, using "generic" health status measures, have been discussed in the literature. Generic HRQOL has the advantage of facilitating the rating of HRQOL of an individual and comparing it across illnesses [9]. The pediatric Quality of Life Inventory™ version 4.0 (PedsQL™) is a generic core scale for ages 2-18 years, which helps to distinguish between healthy children and paediatric patients with acute or chronic health conditions[10]. Stavros Petrou et al. (2007) conducted a study on the health status and health-related quality of life preference-based outcomes of children aged 7 to 9 years with bilateral permanent childhood hearing impairment. They found that children with bilateral permanent childhood hearing impairment had significantly lower single-attribute utility scores in 6 of the 8 attributes of the Health Utilities Index Mark III [11]. It is well-documented that hearing loss has a significant and negative effect on HRQOL. However, the health related quality of life (HRQOL) of hearing-impaired children who also have motor impairment is not well predicted in relation to their normally developing peers. The aims of the present study were threefold: (1) to evaluate the health related quality of life (HRQOL) of deaf primary schoolchildren with motor impairment, (2) to evaluate the health related quality of life (HRQOL) of deaf primary schoolchildren without motor impairment, and (3) to compare the health related quality of life (HRQOL) of deaf children with or without motor impairment with that of normal mainstream schoolchildren.

## 2. Materials and methods

### 2.1 Study background and sample

This study was undertaken as a part of doctoral program. This study was conducted on primary school children aged between 6-11 years, since they can better understand the statements of the questionnaire compared to pre-school children, enabling measurement of the physical, psychosocial, and academic achievements of the children from their own perspectives. The principal caregiver was contacted, and informed consent was obtained from all participants and their parents. For the purpose of comparison with a group of children without hearing impairment, a group of age matched children with normal hearing were identified and assessed using the same measures. Demographic, medical, and audiological data were collected from review of case records of audiologist, family practitioners, speech and language therapists, special teachers and parents. All the hearing impaired children were further assessed by a physiotherapist to determine the motor impairment. Children with any known intellectual disability, major medical disorder, the cognitive, physical, visual or neurological conditions other than sensorineural hearing loss and vestibular impairment were excluded from the study.

### 2.2 Methods

Three groups of 100 children matched for age were recruited for the study. The control group (Group 1) had no history of hearing loss or any other disorders. The two experimental groups (Group 2 & Group 3) consisted of children with hearing impairment alone without motor impairment and children with hearing impairment along with motor impairment respectively. The two experimental groups were recruited from deaf schools.

### 2.3 Procedure

All the participants were asked to answer the PedsQL™ 4.0 questionnaire. Depending on the reading ability of the children, the questionnaire was either read by the children themselves or was presented to them by the examiner through sign language. For each statement in the questionnaire, the children were asked to respond either as "never", "almost never", "sometimes", "often" or "almost always", carrying points 0, 1, 2, 3 and 4, respectively. All the participants responded to the PedsQL™ 4.0 questionnaire anonymously, recording their individual ID number. No expenditure was inflicted on the cases and all the personal records were kept confidential. The study was started after receiving approval from the institutional ethical committee.

### 2.4 Questionnaire

To assess the HRQOL, the PedsQL™ 4.0 questionnaire was used. PedsQL™ 4.0 is a 23 item HRQOL inventory that is comprised of four subdomains: (i) Physical functioning (eight items), (ii) Emotional functioning (five items), (iii) Social functioning (five items), and (iv) School functioning (five items). It is a five-point rating scale. The response scale for each item was ''never'' (0), ''almost never'' (1), ''sometimes'' (2), ''often'' (3), and ''almost always'' (4). Responses were transformed to 100, 75, 50, 25 and 0, respectively, resulting in a scale range of 0-100. The physical health, emotional health, social health, school functioning scores are the mean of the items answered under the physical functioning, emotional functioning, social functioning, and school functioning sub-scales respectively. The total score is the mean value of all the items answered. Scale scores are computed as the sum of the items divided by the number of items answered. Scale scores are not computed if more than a half of items in the scale are missing [9,10]. The PedsQL™ has been used for deaf children [12,13]. Generic HRQOL instrument (PedsQL™ 4.0) enables us to rate the health related quality of life of an individual and compare it across illnesses [9,10]. The items include normative data that allows the comparison of HRQOL of deaf children against normal hearing children.

## 3. Statistical Analysis

The results were analyzed statistically using SPSS software version 16.0. Kruskal-Wallis test was done to compare the differences in three groups of children. Hypothesis was tested with α-level of 0.05. In order to judge the paired comparison, post hoc analysis was done at 95% CI by using the formula K = (d - 0.8)/(N × √N), where d is the difference between rank total of one group and rank total of the other group, and N is the number of subjects.

## 4. Results

**Figure 1:**
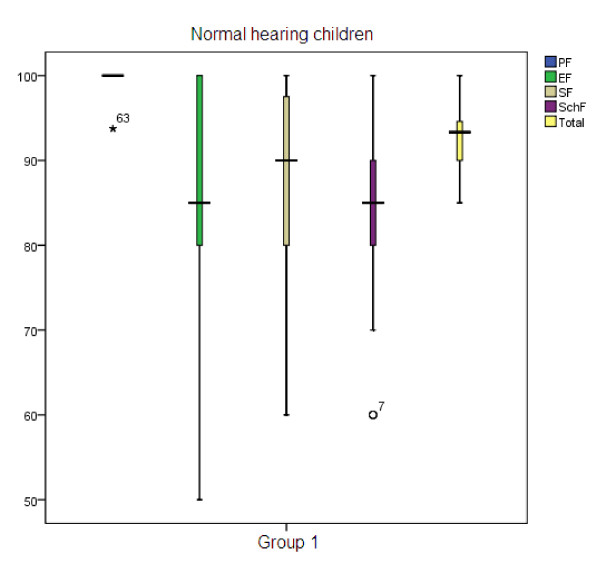
**Group 1 - Normal hearing children**.

**Figure 2:**
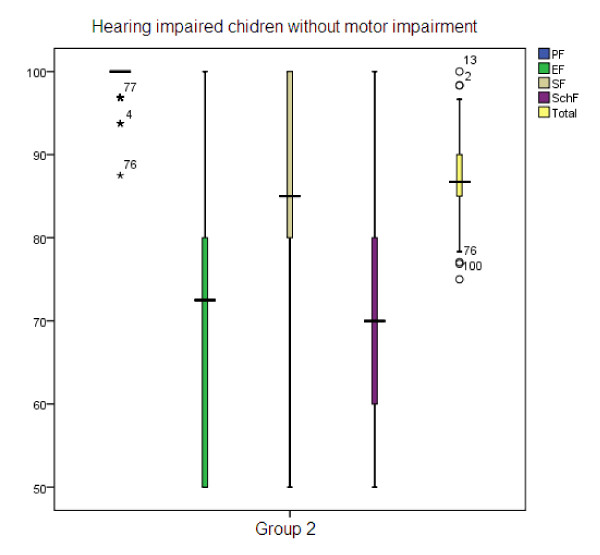
**Group 2 - Hearing impaired children without motor impairment**.

**Figure 3:**
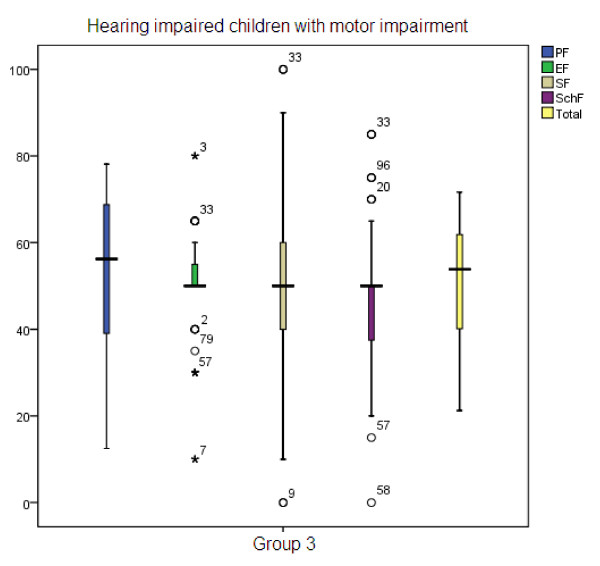
**Group 3 - Hearing impaired children with motor impairment**.

Table 1 illustrates the mean age and gender percentage in three groups. The mean age of participants of group 1, group 2, and group 3 were 8.39, 8.46, and 9.88, respectively. To compare the HRQOL in three groups, we used Kruskal-Wallis test. Significant differences were found in physical health (p < 0.001), emotional health (p < 0.001), social health (p < 0.001), school function (p < 0.001) domains and total score (p < 0.001) (Table 2). Children with normal hearing always scored better. Figure 1 shows the box plot of the scores of normal hearing children reached in the different subscales, with standard deviation. As shown in Table 3, post hoc analysis revealed that there were no significant differences in physical and social health domains between group 1 and group 2, but there were significant statistical differences in all other domains. Furthermore, significant differences were found between other groups. Figure 2 shows the box plot of the scores of children with hearing impairment but without motor impairment reached in the different subscales, with standard deviation. Figure 3 shows the box plot of the scores of children with hearing impairment along with motor impairment in the different subscales, with standard deviation.

**Table 1:** Mean age and percentage of gender

Group	Mean Age	% of Gender	
		Male	Female
Group 1(N^ф ^= 100)	8.39	51	49
Group 2 (N^ф ^= 100)	8.46	59	41
Group 3 (N^ф ^= 100)	9.88	48	52

N^ф^: Number of subjects

**Table 2:** Kruskal-Wallis analysis of HRQOL domains

HRQOL domains/ Scale	Group 1 (N^ф ^= 100)	Group 2 (N^ф ^= 100)	Group 3 (N^ф ^= 100)	Test statistics at 2 df^ג^	
	Mean Rank	Mean Rank	Mean Rank	Chi-square	p-value
Physical health	209.76	191.24	50.50	251.728	p < 0.001
Emotional health	221.38	156.36	73.76	149.422	p < 0.001
Social health	195.16	193.11	63.23	154.566	p < 0.001
School function	228.92	161.12	61.47	191.444	p < 0.001
Total score	231.94	169.06	50.50	225.849	p < 0.001

N**^ф^**: Number of subjects; df^ג^: degrees of freedom

**Table 3:** Post-hoc paired comparisons of Kruskal-Wallis test

HRQOL Domains/Scale	Total Rank G1^§^ (G1 Mean rank × N)	Total Rank G2^¶^ (G2 Mean rank × N)	Total Rank G3^۹^ (G3 Mean rank × N)	d1 (G1-G2 total rank)	d2 (G1-G3 total rank)	d3 (G2 - G3 total rank)	K value (G1&G2)	K value (G1&G3)	K value (G2&G3)
Physical health	20976	19124	5050	1852	15926	14074	1.8512**	15.9252	14.0732
Emotional health	22138	15636	7376	6502	14762	8260	6.5012	14.7612	8.2592
Social health	19516	19311	6323	205	13193	12988	0.2042**	13.1922	12.9872
School function	22892	16172	6147	6720	16745	10025	6.7192	16.7442	10.0242
Total score	23194	16906	5050	6288	18144	11856	6.2872	18.1432	11.8552

G1**^§ ^**: Group 1; G2^¶^: Group 2; G3^۹^: Group 3, ** K value is less than table value (2.89 @ 95% CI)- Insignificant.

## 5. Discussion

The overall results of this study revealed that children with both hearing and motor impairment are associated with significantly increased suboptimal levels of function and significantly lower HRQOL. Children with hearing impairment alone-without any motor involvement-do not show any statistically significant difference in physical and social health scores when compared with their peers with normal hearing. However, there was a statistically significant difference in all four domains as well as the total score of HRQOL when children with hearing impairment and motor involvement were compared with children with hearing impairment without any motor involvement and with normal hearing children.

The finding of the study does not show a significant reduction in physical health in the hearing-impaired group. Annerose Keilmann et al. (2007) conducted a study on the psychological and physical well-being in hearing-impaired children and found that physical well-being was not affected [14]. Mellissa Wake et al. (2010) conducted a study on the effects of slight/mild bilateral sensorineural hearing loss and found no difference in physical functioning between the hearing-impaired group and the group with normal hearing [13]. The study demonstrated a significant reduction in physical health in the group with both hearing and motor impairment when this group was compared with the group with normal hearing and the group with hearing impairment alone. Stavros Petrov et al. (2010) conducted a study on the health status and HRQOL preference-based outcomes of children with bilateral permanent childhood hearing impairment and found a significant difference in the ambulation and dexterity attributes of the Health Utilities Index Mark III [11].

The findings of this study demonstrated a significant reduction in the emotional component results of both the group with hearing-impairment alone and the group with hearing and motor impairment. Wake et al. (2004) found that children with hearing loss achieved significantly lower scores on the emotional component than a normative sample [15]. The result of our study stands in contrast to the studies done by Stavros Petrov et al. (2007) [11], Mellissa Wake et al. (2006) [13], and Annerose Keilmann et al. (2007) [14].

The findings of our study revealed no significant difference in the social component results of the group with hearing impairment alone and the normal peer group. The results were in accordance with the study done by Mellissa Wake et al. (2006) [13], which also did not find any significant difference in the social component in PedsQOL of children with mild hearing impairment and children with normal hearing. However, a significant difference was found between the group with both hearing and motor impairment and the other two groups. Wake et al. (2004) found a significant difference in the social component results of the group with congenital hearing loss when compared with the group with normal hearing [15].

The results of this study showed a significant difference in academic performance in the group with hearing impairment alone and the group with both hearing and motor impairment when compared with the group with normal hearing. The finding contradicts the study done by Mellissa Wake et al. (2006) [13], as they found no significant difference in academic performance between the hearing impaired and normal children.

## 6. Conclusion

In all aspects, children with hearing impairment and motor impairment have shown significantly increased suboptimal levels of function and significantly lower health related quality of life (HRQOL). The reduced physical abilities and diminished health related quality of life of such children must be taken into consideration when making decisions about the appropriate type of service for them.

## Abbreviations

PedsQL™ 4.0: Paediatric quality of life inventory version 4.0; HRQOL: Health related quality of life; PF: Physical functioning; EF: emotional functioning; SF: Social functioning; SchF: School functioning.

## Competing interests

The authors declare that they have no competing interests.

## Authors' contributions

Both the authors contributed to the conception of the study and were involved in writing, revising and approving the final draft of the manuscript.

## Authors' information

- **VR: **PhD Scholar (Rehabilitation), Institute of Rehabilitation Science, Holy Cross College affiliated to Bharathidasan University, Trichy, Tamilnadu, INDIA.

- **FGR: **Assistant Professor, Institute of Rehabilitation Science, Holy Cross College affiliated to Bharathidasan University, Trichy, Tamilnadu, INDIA.
